# New diagnostic algorithm for detection of covert Bow Hunter's Syndrome

**DOI:** 10.7150/ijms.56442

**Published:** 2021-03-19

**Authors:** Luna Kimihira, Takeshi Yoshimoto, Masafumi Ihara

**Affiliations:** 1 Department of Neurology, National Cerebral and Cardiovascular Center, Osaka, Japan.; 2 Department of Cerebrovascular Medicine, National Cerebral and Cardiovascular Center, Osaka, Japan.

**Keywords:** bow hunter's syndrome, covert bow hunter's syndrome, diagnosis, algorithm

## Abstract

Bow hunter's syndrome (BHS) should not be overlooked as a cause of cerebral infarction in the posterior circulation. However, covert BHS, which does not impair blood flow with simple rotation but only at certain angles, may make the diagnosis of BHS difficult. We propose a new algorithm to detect BHS or covert BHS. We recommend that BHS and covert BHS be detected by noninvasive duplex ultrasonography, which will allow for appropriate treatment.

## Introduction

We read with great interest the recent review paper by Miao et al. 1 on the clinical importance of the posterior inferior cerebellar artery in many diseases that result from posterior circulation failure. In the article, the authors mention that termination of the vertebral artery (VA) in the posterior inferior cerebellar artery can cause bow hunter's syndrome (BHS). No agreement has been reached regarding the standard treatment of BHS because of the low number of affected patients.

The incidence of BHS remains unclear because of the limited number of case reports and case series and the lack of interest in BHS in daily clinical practice. Another problem seems to lie in the fact that most studies have reported only the diagnosis and outcomes of BHS, not how BHS can be diagnosed or whether unilateral or bilateral BHS was missed in specific cases. We herein propose a new disease concept called “covert BHS,” stress the importance of various head rotations for ultrasonographic diagnosis of covert BHS, and provide practical guidelines to diagnose BHS and covert BHS in the clinical context.

**Figure 1:**
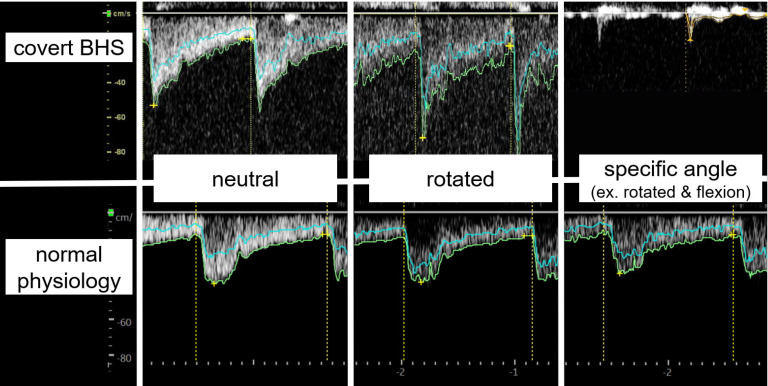
** Doppler ultrasound findings of covert BHS and normal physiology.** In normal physiology, there is no decrease in the EDV at any cervical angle. In covert BHS, however, there is an apparent decrease in the EDV only at specific angles. However, covert BHS shows a typical waveform only by merely rotating the neck horizontally, which is its main difference from common BHS. Abbreviations: EDV, end-diastolic flow velocity; BHS, bow hunter's syndrome.

We define “covert BHS” as BHS in which the blood flow in the VA is impaired only during cervical rotation to a specific angle. Specifically, covert BHS is defined as BHS in which the blood flow of the VA is not interrupted by simple horizontal rotation of the neck and is instead interrupted only by complicated movement of the neck regardless of concurrent subjective neurological symptoms (Figure 1). We previously reported a case involving a young woman who was asymptomatic with insufficient compression by horizontal rotation alone and became symptomatic with more complex movement (i.e., a combination of right flexion and upper rotation) 2. Because various cervical movements may compress the VA, covert BHS cannot be detected by simple cervical rotation alone. Examinations commonly performed to diagnose BHS are duplex ultrasonography, computed tomography angiography, magnetic resonance angiography, and digital subtraction angiography (DSA) 3, 4. Duplex ultrasonography can be performed as a noninvasive and simple screening test, and the definitive diagnosis is obtained by DSA. With proper care and attention, hidden causes of cerebral infarction can be identified using this technique; covert BHS cannot be detected using any other test without utilizing a specific angle. Identifying covert BHS is also essential to determine effective treatment strategies, particularly in patients with bilateral BHS.

In a previous study, 15 of 126 (12%) cases of BHS were of the bilateral type 5. Conservative treatment is the first-line intervention for BHS and can reportedly prevent recurrences 6. Still, BHS occasionally requires surgical treatment when accompanied by repeated severe symptoms. Furthermore, Rastogi et al. 7 reported that among cases in which surgery was the primary treatment approach, 96% of the patients had favorable outcomes, whereas among cases treated by a conservative approach, only 37% of the patients had favorable outcomes. In rare cases of BHS caused by abnormal bone structures, removal of abnormal projections may improve the condition 8, 9. However, the primary surgical procedures are VA decompression and posterior C1-C2 fusion, either of which can be selected in patients with hemodynamic abnormalities caused by unilateral BHS 10. In patients with bilateral BHS, however, bilateral decompression is not recommended because of its high invasiveness; instead, posterior C1-C2 fusion should be selected despite its postoperative inconvenience 3. Extensive ultrasonographic evaluation should be incorporated into the conventional diagnostic procedures for BHS because laterality may substantially affect the choice of surgical procedures to prevent symptom recurrence.

**Figure 2:**
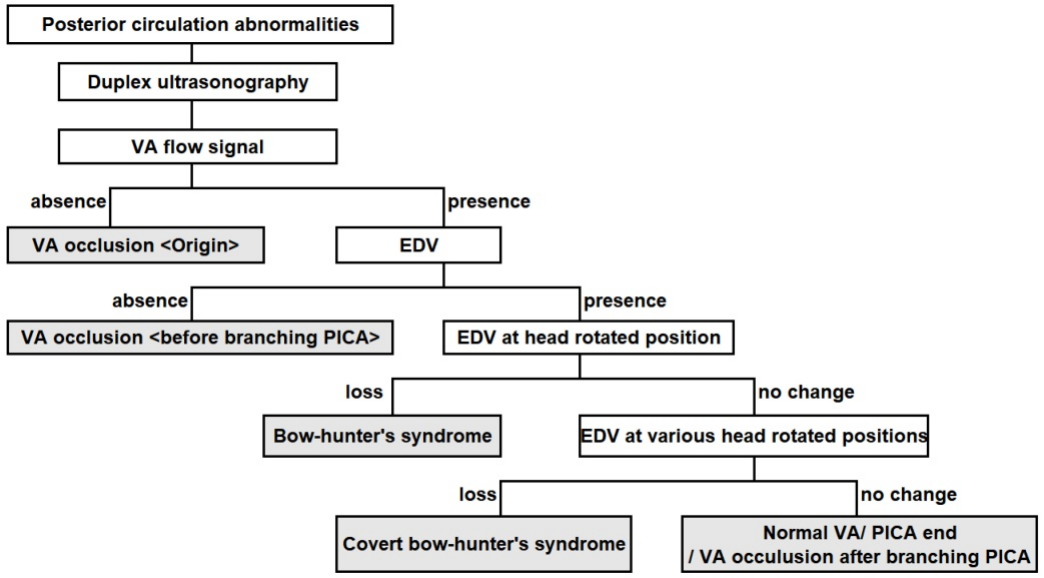
** Ultrasonographic diagnostic chart for bow hunter's syndrome.** Covert BHS is detected as follows. When the EDV of the VA is detectable, then the head should be rotated from side to side. BHS is suspected when the EDV of the VA disappears in a rotated position. When the EDV is maintained in simple rotation, the examiner should continue the evaluation at various head rotation angles. If the EDV is lost at a particular angle, the diagnosis of covert BHS can be confirmed. Abbreviations: BHS, bow-hunter's syndrome; EDV, end-diastolic flow velocity; PICA, posterior inferior cerebellar artery; VA, vertebral artery.

**Figure 3:**
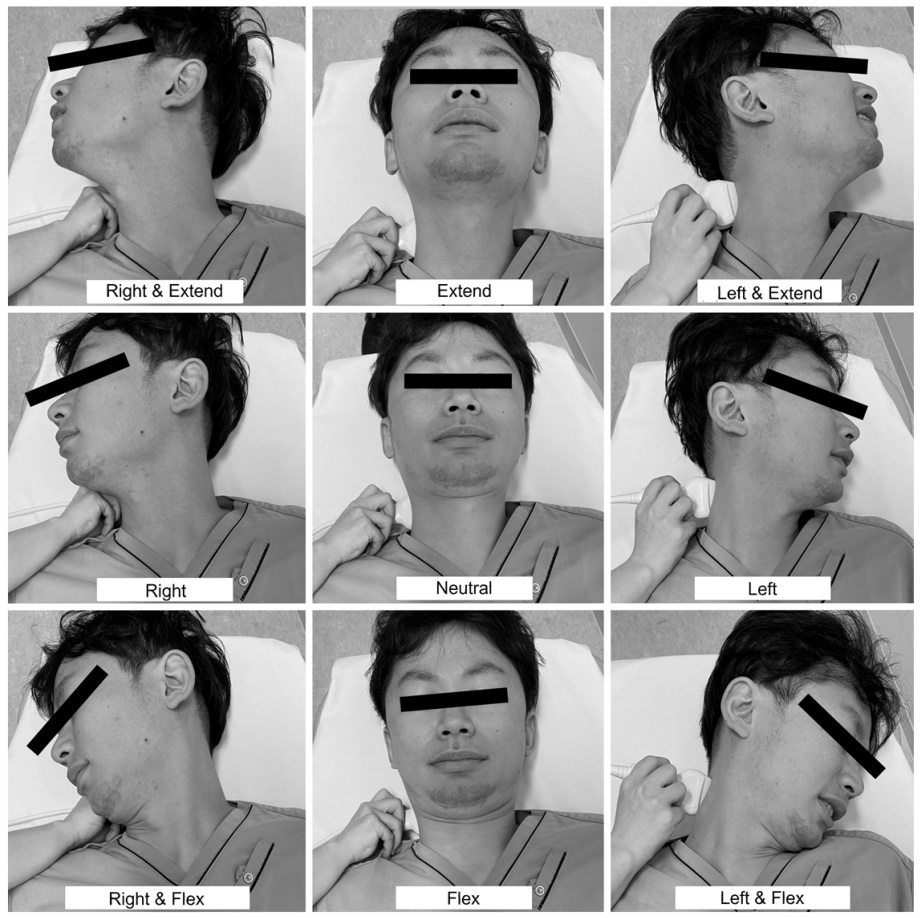
** Nine positions to detect covert bow hunter's syndrome.** The nine preferred angles for routine assessment are the neutral position, right rotated position, right rotated and extended position, right rotated and flexed position, extended position, flexed position, left rotated position, left rotated and extended position, and left rotated and flexed position.

Although Saito et al. 11 proposed a well-known ultrasonographic diagnostic algorithm with which to localize VA occlusion, this algorithm is not sufficient for identifying covert BHS. Disappearance of the end-diastolic flow velocity (EDV) due to cervical rotation reportedly suggests a diagnosis of BHS 3, which should not be missed in patients with posterior circulation stroke of undetermined source. It is necessary to evaluate the EDV in various positions of head rotation to avoid underdiagnosing BHS. Therefore, we created a new algorithm to identify covert BHS by adding head movements to the previous algorithm (Figure 2). If a posterior circulation abnormality is suspected, the VA flow should be evaluated by duplex ultrasonography. Occlusion of the VA origin is suspected when the VA flow signal is absent. In contrast, occlusion of the VA before it branches into the posterior inferior cerebellar artery is suspected when the EDV of the VA is lost in the neutral position. When the EDV of the VA is detectable, then the head should be rotated from side to side. BHS is suspected when the EDV of the VA disappears in a rotated position. In such cases, it is essential to ask the patient about the presence of symptoms such as dizziness. When the EDV is maintained in simple rotation, the examiner should continue the evaluation at various head rotation angles. The nine preferred angles for routine assessment are the neutral position, right rotated position, right rotated and extended position, right rotated and flexed position, extended position, flexed position, left rotated position, left rotated and extended position, and left rotated and flexed position (Figure 3). Instead of randomly turning the head to identify the appropriate neck position, patients should be asked about their usual pillow height and position (a useful question, especially if the patient has a history of stroke while sleeping), type of movement likely to cause the symptoms, and neck position when the symptoms occur. If the EDV is lost at a particular angle, the diagnosis of covert BHS can be confirmed. As mentioned above, BHS is not limited to one side; evaluation of the other side is mandatory. Another important point is that patients with early-onset cerebral infarction in the posterior circulation, especially when vascular comorbidities or cerebrovascular risk factors are absent, should be thoroughly assessed for BHS.

Any clinician with the skills to perform conventional ultrasonography can perform this assessment, and it can be expected to be beneficial with little effort. Although this method is currently used as a screening test for patients with posterior circulation stroke of undermined source, a sufficient number of cases has not yet been accumulated because of the low frequency of BHS and covert BHS. To contribute to the scientific literature, the proposed technique must be verified in a patient with known BHS before surgical treatment to confirm non-inferiority against existing processes. These are the limitations of this proposal. In the future, we plan to examine the differences in the detection rates of BHS, including covert BHS, before and after the introduction of this new algorithm.

The gold standard diagnostic technique for BHS is definitely DSA. However, unless a tentative diagnosis of BHS is given using a screening test with ultrasonography, physicians will not attempt invasive DSA. In other words, we do not intend to replace DSA with ultrasonography or prove the non-inferiority of ultrasonography compared with DSA; instead, we propose a screening algorithm with noninvasive ultrasonography.

In summary, patients with posterior circulation stroke should undergo duplex ultrasonography of the bilateral VAs at various head rotation angles. Proper identification of covert BHS, unilaterally or bilaterally, is clinically significant with respect to determining the most appropriate treatment. We should be careful to avoid missing covert BHS.
